# The Big Three Perfectionism Scale–Short Form: An item response theory analysis of Italian workers

**DOI:** 10.3389/fpsyg.2022.971226

**Published:** 2022-07-22

**Authors:** Andrea Svicher, Alessio Gori, Annamaria Di Fabio

**Affiliations:** ^1^Section of Psychology, Department of Education, Languages, Intercultures, Literatures and Psychology, University of Florence, Florence, Italy; ^2^Section of Psychology, Department of Health Sciences, University of Florence, Florence, Italy

**Keywords:** perfectionism, Big Three Perfectionism Scale–Short Form, item response theory, workers, rigid perfectionism, self-critical perfectionism, narcissistic perfectionism

## Abstract

**Background:**

The present study examined the psychometric properties of the Big Three Perfectionism Scale–Short Form (BTPS-SF) using Rasch and Mokken item response theory (IRT) analyses, which have not previously been applied to the BTPS-SF.

**Materials and methods:**

A total of 401 Italian workers (*M*_age_ = 46.78; SD = 10.1; male = 48.9%; female = 51.1%) completed the BTPS-SF questionnaire. We conducted confirmatory factor analyses of the BTPS-SF and IRT analyses using the generalized partial credit model (GPCM) and Mokken scale analysis. Discrimination and difficulty parameters were calculated. The Loevinger coefficient of scalability was computed. Item characteristic curves (ICC), test information function (TIF), and differential item functioning (DIF) for gender were calculated.

**Results:**

A three-factor solution revealed the best fit. Thus, IRT analyses were performed for each BTPS-SF factor: rigid perfectionism (RP), self-critical perfectionism (SP), and narcissistic perfectionism (NP). All the items showed Loevinger coefficients from medium to strong and discrimination parameters from medium to very high. No DIF for gender was found.

**Conclusion:**

The Big BTPS-SF shows good psychometric properties for Italian workers. Future research is warranted to examine the findings in workers from different countries.

## Introduction

Psychological well-being in the workplace has gained increasing prominence in the research agendas of countries and national and international institutions (Blustein et al., 2019). As a result, various lines of research have begun to expand the knowledge of the psychological variables that impact organizational well-being (Peiró and Tetrick, 2011; Cartwright and Cooper, 2014; Di Fabio, 2017). In particular, industrial-organizational (I/O) psychology scholars have begun to analyze the contribution of perfectionism to workplace well-being (e.g., Sirois and Molnar, 2016; Harari et al., 2018; Ocampo et al., 2020).

Perfectionism is a multidimensional personality trait that encompasses high personal standards and critical evaluations of oneself and others (Frost et al., 1990; Hewitt and Flett, 1991). Frost et al. (1990) identified five dimensions of perfectionism: concern over mistakes, personal standards, parental expectations, parental criticism, and doubts about actions. Hewitt and Flett (1991) described three dimensions of perfectionism—self-oriented, other-oriented, and socially prescribed perfectionism—which they further aggregated into two forms: perfectionistic concerns and perfectionistic strivings (Stoeber and Otto, 2006). More recently, Smith et al. (2016) proposed a model composed of rigid perfectionism (RP), self-critical perfectionism (SCP), and narcissistic perfectionism (NP).

Previous studies have shown that perfectionistic concerns negatively affected employee well-being (Kanten and Yesıltas, 2015; Hill and Curran, 2016) and were associated with occupational fatigue (Magnusson et al., 1996; Childs and Stoeber, 2012; Flaxman et al., 2012), stress (Dunkley et al., 2014; Mandel et al., 2018), and burnout (Hill and Curran, 2016). Additional findings suggested that self-oriented perfectionism was positively associated with performance anxiety (Kobori et al., 2011) and depressive symptoms (Gluschkoff et al., 2017). Data obtained via daily longitudinal methods highlighted perfectionistic concerns linked with poorer sleep quality and work day functioning (Flaxman et al., 2018). Moreover, qualitative findings reported that perfectionism was the main factor hindering workers from returning to work after experiencing burnout (Noordik et al., 2011). Perfectionism has been shown to negatively affect the relational aspects of work (Fairlie and Flett, 2003; Dunkley et al., 2014; Mandel et al., 2018). Employees with high levels of socially prescribed perfectionism were more likely to report worsened relationships with their coworkers and supervisors (Fairlie and Flett, 2003) and avoid relationships and coworkers’ support (Mandel et al., 2018). On the contrary, a higher level of team friendship in the workplace weakened the positive association between perfectionistic concerns and job burnout (Chang et al., 2016). Other results showed an association between perceived justice in the workplace and perfectionism, highlighting that workers with low levels of justice perceptions and high perfectionistic concerns displayed counterproductive work behaviors (Beauregard, 2014). Similarly, when perceived justice in the workplace was found to be low, the moderating effect of employee’s socially prescribed perfectionism between interactional justice and organizational citizenship behavior was found to be significant (Kim et al., 2022). In addition, studies have found that perfectionistic concerns were positively associated with higher work–family conflict (Mitchelson, 2009; Deuling and Burns, 2017), with men that are more prone to show maladaptive levels of perfectionism in association with work-family conflict (Ekmekci et al., 2021). Furthermore, self-oriented perfectionism (Stoeber et al., 2013) and perfectionistic strivings (Stoeber and Damian, 2016; Spagnoli et al., 2021b) and concerns (Stoeber and Damian, 2016; Spagnoli et al., 2021a) have been found to be positively associated with workaholism. Moreover, other-oriented perfectionism in leaders was found to be associated with monitoring behaviors and highlighted as a barrier to building trusting relationships (Otto et al., 2021). Other results investigated the effects of the interaction between managers’ perfectionism and employees’ perfectionism on work addiction, revealing that employees’ socially prescribed perfectionism and work addiction was strongest when a manager was perceived to be addicted to work (Morkevičiûtė and Endriulaitienė, 2022).

Only a handful of studies have investigated perfectionism in Italian workers (Spagnoli et al., 2021a,b), for example, showing that perfectionism was positively associated with both positive and negative forms of heavy work investment (Mazzetti et al., 2020). However, no research has applied Smith et al.’s (2016) model, yet. To expand the knowledge of the multidimensional approach to perfectionism, Smith et al. (2016) recently released the Big Three Perfectionism Scale (BTPS). BPTS is a 45-item self-report measure that assesses the above-mentioned three dimensions of perfectionism (i.e., RP, SCP, and NP) to provide a fine-grained analysis of the construct (Smith et al., 2016). Using this framework, Feher et al. (2019) subsequently created a brief version of the BTPS, known as the Big Three Perfectionism Scale–Short Form (BTPS-SF), and Di Fabio et al. (2018) adapted it to the Italian context. Previous studies on the BTPS-SF have involved university students (Di Fabio et al., 2018; Feher et al., 2019) and have relied on classic test theory (CTT). As an alternative to CCT, item response theory (IRT; e.g., Van der Linden, 2018) is a broadly used psychometric approach that includes scaling of latent variables via the evaluation of homogeneity coefficients (Sijtsma and Verweij, 1992), the calculation of reliability, taking into account different levels of abilities (Embretson and Reise, 2000), and the evaluation of item bias through difficulty and discrimination parameters (Embretson and Reise, 2000). Thus, IRT is a promising approach to conducting a fine-scale analysis of a self-report tool, and it is also in line with the accountability perspective, which encourages researchers to use evidence-based methodologies to ensure a balance in terms of cost-effectiveness (Whiston, 1996, 2001. The use of short-form questionnaires in organizations could be a promising strategy for decreasing the costs of research intervention while ensuring reliability (Whiston, 1996, 2001).

Therefore, this study aims to test the psychometric properties of the BTPS-SF in Italian workers by applying IRT models. We evaluated the homogeneity of the BTPS-SF items and dimensions to test whether the total summed item scores provided satisfactory statistics. IRT reliability statistics were calculated to examine the extent to which the BTPS-SF is a reliable tool for workers with different levels of rigid, self-critical, and narcissistic perfectionism. Item-level measurement bias due to gender-related differences was assessed to analyze its contribution to the BTPS-SF items.

## Materials and methods

### Participants and procedure

The study was conducted with 401 workers employed at private and public organizations in central-southern Italy (males = 48.9%; females = 51.1%; mean age = 46.78 years, SD = 10.1; age range: 27–65 years). Participants were workers employed at different public and private organizations recruited voluntarily from their organizations, who granted permission for research in their setting. The participants were predominantly white Italian regular workers who chose to participate in the study voluntarily. General information about the aims of the study was communicated to the participants in advance. The workers provided their informed written consent, and the research was carried out according to the ethical standards of Italian law (Law Decree DL-196/2003) and the European Union General Data Protection Regulation (EU 2016/679).

### Measurement

#### Big Three Perfectionism Scale–Short Form – Italian version

The Italian version of the Big Three Perfectionism Scale–Short Form (BTPS-SF; Di Fabio et al., 2018) is a self-report questionnaire that assesses three dimensions of perfectionism: rigid perfectionism (demanding flawless performance from the self; example of item: “I have a strong need to be perfect”); self-critical perfectionism (concerns about imperfect performance and propensity to be severely self-critical when performance is not perfect; example of item: “The idea of making a mistake frightens me”); and narcissistic perfectionism (demanding perfection from others in a grandiose, hypercritical, and entitled way; example of item: “I get frustrated when other people make mistakes”). The Italian version of the BTPS-SF was developed by Di Fabio et al. (2018) starting from the English version of the 45-item BPTS (Smith et al., 2016). They identified a satisfactory three-factor structure (CFI = 0.91; TLI = 0.90; Cronbach’s alphas ranging from 0.83 to 0.89) with each factors enclosing six items ranked on a 5-point Likert scale (From 1 = strongly agree to 5 = strongly disagree). Subsequently, Feher et al. (2019) derived the English BTPS-SF. They retained a three-factor structure and selected the 16 items with the highest loadings (ranging from 0.43 to 0.83) and minimal or no cross-loadings on other factors. This structure showed the best fit compared with a one-factor structure (Feher et al., 2019).

### Data analysis

R Studio for Macintosh (Version 1.3.959) was used to analyze the data. The specific R package used for each analysis is provided in each subsection.

#### Factor analysis

We conducted a confirmatory factor analysis (CFA) to compare two different models of the BTPS-SF in line with Feher et al. (2019) and to expand results of Di Fabio et al. (2018). The first model was a one-factor model reflecting the view of researchers who conceive perfectionism as a unidimensional construct (e.g., Shafran et al., 2002). The second model was a three-factor model that reflects the three dimensions of rigid, self-critical, and narcissistic perfectionism (six items for each dimension) (Di Fabio et al., 2018; Feher et al., 2019). CFA was implemented by applying the mean- and variance-adjusted weighted least square estimation (WLSMV). Model fit was evaluated via the comparative fit index (CFI), the Tucker–Lewis Index (TLI), and the root mean square error of approximation (RMSEA). CFI and TLI values greater than 0.97 indicated a good fit, whereas values ranging from 0.95 to 0.97 indicated an acceptable fit. RMSEA values were evaluated as follows: good (≤0.05), adequate (0.05–0.08), mediocre (0.08–0.10), and unacceptable (>0.10) (Schermelleh-Engel et al., 2003). The lavaan 0.6-9 and SemPlot 1.1.2 R packages were used.

#### Item response theory analysis

Item response theory GPCM analyses were run using marginal maximum likelihood. Mean-square infit and outfit statistics were used to evaluate the fit of BTPS-SF items under the GPCM model (values close to 1.00 indicated a good fit). Furthermore, the root-mean-square deviation (RMSD) was run as an additional index of item-fit statistics. RMSD values <0.05 suggested a good item fit. The IRT GPCM model provides, for each item, discrimination (a) and difficulty (b) parameters as well as item thresholds (τ). The discrimination parameter (a) was used to evaluate whether each item could discriminate between subjects with different levels of perfectionism (Reise and Henson, 2003). Values <0.64 indicate unacceptable discrimination, values between 0.65 and 1.34 indicate moderate discrimination, values between 1.35 and 1.69 indicate high discrimination, and values ≥1.70 indicate very high discrimination (Baker, 2001). The difficulty parameter (b) was used to estimate the difficulty of each item (Muraki, 1992). Values close to zero represent medium difficulty, negative (b) values indicate less difficulty, and positive (b) values represent more difficulty (Baker, 2001; Reise and Henson, 2003). Given that items in the BTPS-SF are ranked on a 5-point Likert scale, they have four item thresholds (i.e., τ_1_, τ_2_, τ_3_, and τ_4_). Each threshold indicates the measured level of perfectionism at which participants have a 50/50 chance of endorsing one or the other Likert scale option (Muraki, 1992). The test information function (TIF) was run to evaluate reliability. The formula applied was as follows: 1 minus the inverse of the total information value [*r* = 1−(1/I)] (Embretson and Reise, 2000). TIF values >3.30 (i.e., *r* = 0.70) indicated good reliability (Cappelleri et al., 2014). The TAM 4.0-16 package was used. Lastly, we investigated differential item functioning (DIF), a type of item-level bias that exists when an item has different measurement properties in the construct measured (Edelen et al., 2006). DIF was used to investigate whether gender (male vs. female) might affect responses to the BTPS-SF. The DIF for gender was conducted using the Lordif R package, which uses a logistic ordinal regression approach based on IRT-based trait scores and iterative purification. McFadden’s R^2^ (McFadden, 1987) was used to identify items with DIF. A value of *R*^2^ < 0.15 indicated negligible DIF (Choi et al., 2011).

#### Reliability analysis

The reliability of the BTPS-SF was assessed using Cronbach’s alpha (α), the Sijtsma and Molenaar (1987) rho (ρ) coefficient, the IRT GCPC expected *a posteriori* (EAP) trait scores index (Adams, 2005), and the Omega hierarchical (ωH) coefficient (McDonald, 1999). Values of α and ρ > 0.70 and an EAP > 0.74 indicate good reliability (Sijtsma and Molenaar, 1987; Nunnally and Bernstein, 1994; Adams, 2005). Values of ω_H_ > 0.70 indicate that a general factor determines the systematic variance of a scale, excluding the possible presence of minor factors underlying unit-weighted total scale scores (Rodriguez et al., 2016). The Psych 2.2.5, TAM 4.0-16, and Mokken 3.06 R packages were used.

## Results

### Factor analysis

**FIGURE 1:**
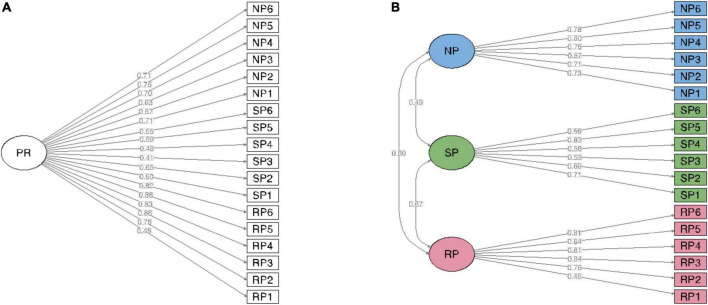
The Big Three Perfectionism Scale–Short Form: Confirmatory factor analysis with weighted least squares means and variance adjusted (WLSMV) estimation. **(A)** One-factor model. **(B)** Three-factor model (*n* = 401). PR, Perfectionism; SP, Self-critical perfectionism; NP, Narcissistic perfectionism.

The three-factor model showed a moderately good fit to the data [χ^2^(df) = 326.61 (132); RMSEA = 0.061 (95% CI = 0.052–0.069); CFI = 0.987; TLI = 0.985], whereas the one-factor model showed a mediocre fit [χ^2^(df) = 668.69 (135) RMSEA = 0.099 (95% CI = 0.092–0.107); CFI = 0.965; TLI = 0.960]. A statistical comparison between the two models revealed that the three-factor solution had the best fit [Δχ^2^(df) 123.2 (3); *p* < 0.001]. Thus, the multidimensional three-factor model, reflecting rigid, self-critical, and narcissistic perfectionism, was retained as the most empirically parsimonious (Figure 1). Therefore, one IRT analysis was performed for each dimension (i.e., RP, SCP, and NP).

### Item response theory analysis

Table 1 shows the results of the IRT Mokken scale analysis. All the items showed adequate homogeneity, with the Mokken coefficient of scalability ranging from medium (SP2: “I have difficulty forgiving myself when my performance is not flawless”) to strong (RP3: “I always need to be aiming for perfection to feel ‘right’ about myself”). Similarly, each BTPS-SF dimension (i.e., RP, SCP, and NP) had strong homogeneity, indicating that the summed total scores of all three dimensions were sufficient statistics (Table 1). Table 1 also displays the results of the IRT GPCM analyses. RMSD values, as well as infit and outfit mean-square statistics, showed good values (RMSD < 0.05; infit/outfit near 1.00); thus, all items fit the GPCM model (Table 1). The results of the IRT GPCM analyses illustrated that the discrimination parameters of all items enclosed in each dimension (i.e., RP, SCP, and NP) ranged from medium to very high (Table 1). Lastly, all items included in the BTPS-SF showed that the item thresholds proceeded from less to more difficulty, thus reflecting the ordered categorical feature of the 5-point Likert scale (Table 1).

**TABLE 1:** The Big Three Perfectionism Scale–Short Form: Generalized partial credit model (GPCM) and Mokken scale analyses (*n* = 401).

	Infit	Outfit	*a*	*b*	τ_1_	τ_2_	τ_3_	τ_4_	RMSD	H_iJ_ (SE)
**RP1.** I strive to be as perfect as possible	1.02	0.97	0.94	−0.90	−1.08	−0.77	0.22	1.63	0.05	0.56 (0.03)
**RP2.** I have a strong need to be perfect	1.01	1.00	1.78	0.11	−0.63	−0.61	0.14	1.10	0.04	0.70 (0.02)
**RP3.** I always need to be aiming for perfection to feel “right” about myself	0.98	0.93	2.75	0.13	−0.61	−0.44	−0.05	1.10	0.04	0.75 (0.02)
**RP4.** I could never respect myself if I stopped trying-to achieve perfection	1.00	0.95	2.24	0.34	−0.70	−0.62	0.08	1.24	0.03	0.72 (0.02)
**RP5.** I never settle for less than perfection from myself	0.99	0.97	2.25	0.49	−0.77	−0.63	0.18	1.22	0.03	0.73 (0.02)
**RP6.** Striving to be as perfect as possible makes me feel worthwhile	1.00	1.05	2.19	0.23	−0.64	−0.50	0.04	1.10	0.04	0.72 (0.02)
**Rigid perfectionism (RP) total score**										**0.70 (0.02)**
**SP1.** People are disappointed in me whenever I don’t do something perfectly	0.99	1.02	0.94	0.16	−1.13	−0.78	0.25	1.66	0.04	0.54 (0.03)
**SP2.** I have difficulty forgiving myself when my performance is not flawless	1.00	1.02	0.65	0.60	−1.43	−1.20	0.58	2.05	0.05	0.44 (0.04)
**SP3.** I am never sure if I am doing things the correct way	1.02	1.00	1.39	0.57	−1.31	−0.29	0.38	1.22	0.04	0.55 (0.03)
**SP4.** I have doubts about everything I do	1.01	1.02	1.92	0.73	−1.12	−0.56	0.47	1.21	0.03	0.58 (0.03)
**SP5.** The idea of making a mistake frightens me	0.99	0.98	1.62	0.56	−0.79	−0.70	0.04	1.45	0.03	0.60 (0.03)
**SP6.** I feel uncertain about most things I do	1.01	0.98	2.35	0.75	−1.07	−0.34	0.24	1.17	0.03	0.59 (0.03)
**Self-critical perfectionism (SP) total score**										**0.55 (0.02**)
**NP1.** I am the absolute best at what I do	1.01	1.02	1.20	0.89	−0.46	−0.73	0.32	0.87	0.04	0.60 (0.03)
**NP2.** I am entitled to special treatment	1.00	1.04	1.45	1.03	−0.80	−0.47	0.28	0.98	0.03	0.63 (0.03)
**NP3.** Other people secretly admire my perfection	1.01	1.02	1.29	0.95	−0.64	−0.63	0.54	0.73	0.04	0.59 (0.03)
**NP4.** I expect those close to me to be perfect	1.03	1.04	2.03	0.98	−0.97	−0.23	0.19	1.01	0.04	0.61 (0.03)
**NP5.** I get frustrated when other people make mistakes	1.00	1.03	1.94	0.94	−0.89	−0.58	0.35	1.11	0.03	0.64 (0.02)
**NP6.** Everything that other people do must be flawless	1.02	0.92	3.15	1.09	−1.01	−0.36	0.44	0.93	0.02	0.67 (0.02)
**Narcissistic perfectionism (NP) total score**										**0.62 (0.02**)

Infit/Outfit, Mean-square infit and outfit statistics; a, Discrimination parameter; b, Difficulty parameter; tau, Trait level; RMSD, Root-mean-square deviation item-fit statistic; Hij, Mokken coefficient of scalability.

**FIGURE 2:**
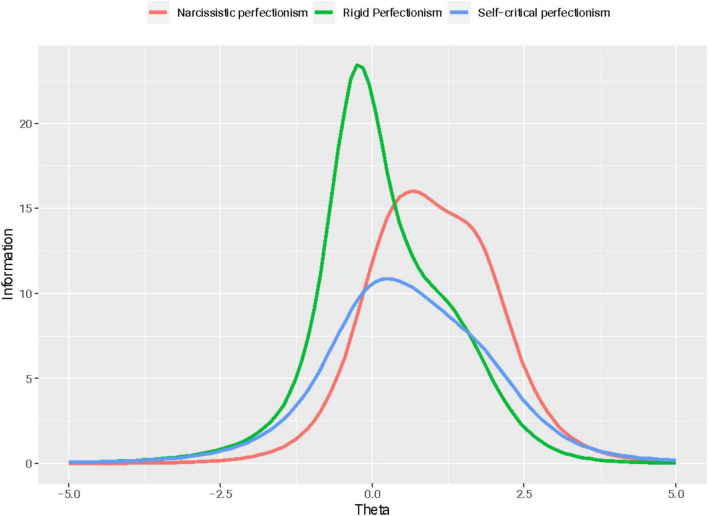
The Big Three Perfectionism Scale–Short Form: Test information functions (GPCM Model) (*n* = 401).

Figure 2 reports the test information functions for the three BTPS-SF dimensions. The TIF curve of rigid perfectionism has its peak value at theta −0.05 (*I* = 20.72), with a high value of reliability between θ = −1.46 (low rigid perfectionism) (*I* = 3.45; *r* = 0.71) and θ = 2.17 (very high rigid perfectionism) (*I* = 3.75; *r* = 0.73). The TIF curve of self-critical perfectionism has its peak value at theta 0.25 (*I* = 10.84) and high reliability, ranging from θ = −1.26 (low self-critical perfectionism) (*I* = 3.36; *r* = 0.70) and θ = 2.47 (very high self-critical perfectionism) (*I* = 3.39; *r* = 0.70). Narcissistic perfectionism showed a TIF curve with a peak corresponding to a theta value of 0.66 (*I* = 16.03) and high reliability ranging from θ = −0.76 (low/medium narcissistic perfectionism) (*I* = 3.66; *r* = 0.72) to θ = 2.78 (very high self-critical perfectionism) (*I* = 3.62; *r* = 0.72). Overall, all three dimensions showed excellent reliability, ranging from low and low/medium to a very high level of the measured traits (i.e., RP, SCP, and NP).

The DIF results illustrated that no items for any dimension were flagged for this criterion (i.e., no single item had a McFadden’s *R*^2^ > 0.15). Thus, the BTPS-SF revealed no DIF related to gender for any of the items. This indicates that the possible differences in item scores due to gender were not due to bias in item functioning.

### Reliability analysis

Table 2 illustrates the statistics used to explore the score reliability of the three BTPS-SF dimensions. All three dimensions showed excellent Cronbach’s alphas (α ranged from 0.86 to 0.92). Consistently, all three dimensions had a high omega hierarchical (ω_H_ from 0.76 to 0.88), indicating the absence of minor factors. The IRT GPCM expected *a posteriori* (EAP) trait score, as well as the IRT Mokken-Molenaar and Sijtsma ρ, showed excellent values (EAP from 0.85 to 0.90 and ρ from 0.83 to 0.87).

**TABLE 2:** The Big Three Perfectionism Scale–Short Form: Indexes of reliability for each dimension (*n* = 401).

BTPS-SF dimension	α	ω_H_	EAP	ρ
Rigid perfectionism (RP)	0.92	0.88	0.90	0.87
Self-critical perfectionism (SP)	0.86	0.76	0.87	0.83
Narcissistic perfectionism (NP)	0.90	0.87	0.85	0.87

α, Cronbach’s alpha; ω_H_, McDonald’s Hierarchical Omega; EAP, expected a posteriori (EAP) trait scores estimated from the GPCM IRT; ρ, Molenaar and Sijtsma rho statistic estimated from the Mokken IRT analysis.

## Discussion

The BTPS-SF showed the best fit for a three-factor solution, reflecting the three dimensions of perfectionism: rigid perfectionism, self-critical perfectionism, and narcissistic perfectionism. This finding is consistent with Smith et al.’s (2016) model and in line with the results of Di Fabio et al. (2018) and Feher et al. (2019).

The Mokken analysis showed that all items in the BTPS-SF had an adequate value of homogeneity. Similarly, the total scores for rigid perfectionism, self-critical perfectionism, and narcissistic perfectionism were sufficient statistics. Since this is the first study to evaluate the BTPS-SF using Mokken analysis, we are not able to compare the findings with the literature. However, the lowest level of homogeneity, even though acceptable, which was shown by item SP2 (“I have difficulty forgiving myself when my performance is not flawless”), could be explained by the fact that in the organizational environment, job performance is “one of the most emotionally charged activities” in working life (Narcisse and Harcourt, 2008). Thus, in the workplace, this item could slightly overlap with traits related to conscientiousness or striving for excellence (Gaudreau, 2018), rather than maladaptive self-critical perfectionism. Furthermore, the IRT GPCM analyses reported that the discrimination parameters of all items ranged from medium to very high and that item thresholds were correctly ordered from less to more difficult. This confirms the excellent psychometric properties observed by Di Fabio et al. (2018) and Feher et al. (2019).

Reliability measured via the TIF showed high reliability from low and low/medium to a very high level of the measured traits (i.e., RP, SCP, and NP). Again, there are no previous results on this topic, since this is the first study that has applied IRT GPCM to BTPS-SF. However, these findings suggest that BTPS-SF is a trustworthy tool for workers with high rigid, self-critical, and narcissistic perfectionism and is capable of detecting those traits that may be more likely to negatively affect the well-being of workers (Sirois and Molnar, 2016; Harari et al., 2018; Ocampo et al., 2020). Reliability measured via IRT and CCT indexes showed excellent values consistent with those obtained by Di Fabio et al. (2018) and Feher et al. (2019).

The DIF BTPS-SF test showed no statistically significant differences in the comparison between males and females. These findings are consistent with previous research that showed that perfectionism in the workplace is invariant across genders (Ocampo et al., 2020).

The current research has several limitations. First, it enrolled workers only from south-central Italy, thus restricting the generalizability of our findings. However, the homogeneous population and sample size (i.e., 401 subjects) (Smith et al., 2008) allow for accurate IRT parameter estimates (Edelen and Reeve, 2007). A comparison among cross-cultural samples is warranted (Edelen and Reeve, 2007), and future studies are needed to explore the findings in workers from different countries. However, our study also has strength to be highlighted. Perfectionism in work contexts is studied above all as an individual variable that modifies organizational perceptions and outcomes being associated with negative work-related outcomes (e.g., Harari et al., 2018; Ocampo et al., 2020). Thus, the assessment of perfectionism at various organizational levels could be promising to prevent or monitor the insurgence of high levels of perfectionism that could negatively impact organizational performances. Furthermore, the assessment of perfectionism could be promising from a healthy business point of view (Di Fabio, 2017). This perspective is focused on the maintenance, promotion, and development of well-being of workers and organizations, taking also into account aspects that could worsen workers’ well-being. Consistently with a healthy business perspective (Di Fabio, 2017), factors that impact workers’ well-being could be ameliorate by implementing preventive strength-based actions (Di Fabio and Peiróì, 2018; Di Fabio and Saklofske, 2021) also at the primary level of intervention (Di Fabio and Kenny, 2019).

## Conclusion

In brief, the Italian version of the BTPS-SF has a three-factor structure, good homogeneity, good discriminative power, and excellent reliability. Thus, the BTPS-SF is a valuable instrument that can be used in practice and research to assess rigid, self-critical, and narcissistic perfectionism in Italian workers.

## Data availability statement

The raw data supporting the conclusions of this article will be made available by the authors, without undue reservation.

## Ethics statement

The studies involving human participants were reviewed and approved by the Ethics Committee of the Integrated Psychodynamic Psychotherapy Institute (IPPI). The patients/participants provided their written informed consent to participate in this study.

## Author contributions

ADF conceptualized the manuscript, supervised, and tutored AS. AS and AG reviewed, edited, and wrote all the draft of the manuscript. AS wrote the first draft of the manuscript and ran the statistical analyses. ADF and AG reviewed, edited, and wrote the final draft of the manuscript. All authors contributed to the article and approved the submitted version.

## Conflict of interest

The authors declare that the research was conducted in the absence of any commercial or financial relationships that could be construed as a potential conflict of interest.

## Publisher’s note

All claims expressed in this article are solely those of the authors and do not necessarily represent those of their affiliated organizations, or those of the publisher, the editors and the reviewers. Any product that may be evaluated in this article, or claim that may be made by its manufacturer, is not guaranteed or endorsed by the publisher.
